# Effects of fucoidan supplementation on inflammatory and immune response after high-intensity exercise

**DOI:** 10.1080/15502783.2023.2224751

**Published:** 2023-06-18

**Authors:** Bridget A. McFadden, Caroline S. Vincenty, Alexa J. Chandler, Harry P. Cintineo, Blaine S. Lints, Gianna F. Mastrofini, Shawn M. Arent

**Affiliations:** a University of South Carolina, Department of Exercise Science, Columbia, SC, USA; b Queens College, City University of New York, Department of Family, Nutrition, and Exercise Sciences, New York, NY, USA; c Lindenwood University, Department of Kinesiology, Saint Charles, MO, USA

**Keywords:** Marine algae supplement, recovery, peak power, cytokines

## Abstract

**Introduction:**

High-intensity exercise (HIE) can damage the musculotendon complex and impact the immune response, resulting in post-exercise inflammation. Sufficient rest and recovery will improve muscular resilience against future damaging bouts; however, HIE with minimal durations of rest is common in athletic competitions that facilitate persistent inflammation and immune dysregulation. Fucoidans are fucose-rich sulfated polysaccharides with demonstrated anti-inflammatory and pro-immune responses. Fucoidans may improve inflammation and immune responses, which may prove beneficial for individuals who regularly engage in repeated HIE. The research purpose was to investigate the safety and efficacy of fucoidans on inflammatory and immune markers following HIE.

**Methods:**

Eight male and eight female participants were randomized into a double-blind, placebo-controlled, counterbalanced, crossover design study and supplemented with 1 g/day fucoidan from *Undaria pinnatifida* (UPF) or placebo (PL) for 2 weeks. Supplementation periods concluded with HIE testing, followed by 1 week of washout. HIE involved one > 30 s Wingate anaerobic test (WAnT) and eight 10 s WAnT intervals. Blood was drawn pre-exercise, immediately post-exercise, 30 min, and 60 min post-exercise to assess immune and inflammatory markers. Blood markers, peak power (PP), and mean power (MP) were analyzed using a 2 (condition) × 4 (time) design. Significance was set at α = .05.

**Results:**

A time-by-condition interaction was observed for interleukin-6 (*p* = .01) and interleukin-10 (*p* = .008). Post hoc analysis revealed greater interleukin-6 and interleukin-10 concentrations at 30 min post HIE with UPF supplementation (*p* = .002 and *p* = .005, respectively). No effects of condition were observed for all blood markers or performance outcomes with UPF supplementation (*p* > .05). Main effects of time were observed for white blood cells, red blood cells, red cell distribution width, mean platelet volume, neutrophils, lymphocytes, monocytes, eosinophils, basophils, natural killer cells, B and T-lymphocytes, CD4 and CD8 cells (*p* < .05).

**Discussion:**

No adverse events were reported throughout the study period, indicating a positive safety profile of UPF. While notable changes in biomarkers occurred up to 1 hr post HIE, few differences were observed between supplementation conditions. There did appear to be a modest effect of UPF on inflammatory cytokines potentially warranting further investigation. However, fucoidan supplementation did not influence exercise performance.

## 1. Introduction

An acute bout of exercise can cause structural damage to the musculotendon complex that necessitates repair following the cessation of the session [1]. The mechanical action of the myocytes can disrupt sarcomere integrity by stretching and manipulating the myofibrils beyond the overlapping filaments [1]. This can lead to disrupted structural components and potential rupture of the cell membranes that may result in unfavorable reductions in strength and the manifestation of inflammation and muscular soreness in the days following exercise [1,2]. Exercise-induced muscular damage (EIMD) is the outcome of the working musculature and connective tissues that are systematically damaged throughout an acute bout of exercise. The severity of EIMD depends on exercise intensity, duration of exercise, and mode of exercise [1–4]. Higher intensities and longer durations of exercise will incur greater damage to the working musculature [1,3,5]. Repeated bouts of exercise with sufficient recovery can induce favorable adaptions of the trained muscles, which may lead to physiological and functional resilience against future EIMD [1,2,6]. However, when sufficient recovery periods are not available, less physiological disruption following high-intensity exercise (HIE) may enable a quicker return to optimal performance. This may be particularly beneficial for individuals who engage in activities that require repeated bouts of HIE, such as scheduled athletic competitions or tactical operations.

Skeletal muscle damage will activate the immune system to help control the damage, clear cellular debris, and initiate repair of the damaged tissues [3]. The magnitude of immune activation correlates with the severity of EIMD, with greater leukocyte infiltration and inflammatory-related cytokine presence observed in longer durations and higher intensities [1–4]. Following HIE, leukocytes, like neutrophils, accumulate locally in the damaged tissue where they secrete cytokines [3,7]. Additionally, lymphocytes become activated and can accumulate, playing an important role in the inflammatory response. These cells are identified by the cluster of differentiation (CD) membrane receptors, such as T-lymphocytes (CD3), B-lymphocytes (CD19), and natural killer cells (CD16 + CD56), along with CD4 cells, which participate in cytokine signaling and CD8 cells, which act to destroy cells infected with viruses [7].

Cytokines can act in both a pro- and anti-inflammatory manner. For example, interleukin-1β (IL-1β) is pro-inflammatory and acts to recruit more leukocytes to the damaged cells [8]. Other cytokines such as interleukin-10 (IL-10) act as anti-inflammatory agents to reduce leukocyte secretion of pro-inflammatory cytokines and increase phagocytic activity of macrophages [9]. Where IL-1β will be secreted following moderate-intensity exercise (MIE) and in a dose-dependent manner, IL-10 is predominantly secreted following HIE [3] and acts to mediate the pro-inflammatory pathway [10,11]. In addition to leukocyte secretion of cytokines, the working muscle can locally secrete cytokines such as interleukin-6 (IL-6) [2,3,10,12]. HIE can quickly deplete the working musculature of glycogen, which will stimulate IL-6 secretion to activate hepatic glycogenolysis and lipolysis [12,13]. This increases glucose availability for the working musculature from extracellular sources. Following exercise, IL-6 initially acts in a pro-inflammatory manner to recruit more immune cells toward the damaged tissues and plays a role in sensitizing local nociceptors to augment the pain response following EIMD [2,14]. IL-6 then has a later role to help mediate the inflammatory response by initiating production of IL-10 and acute-phase proteins in the liver, like C-reactive protein, which work to shift leukocyte production away from pro-inflammatory actions [2,12]. In this way, IL-6 works in an “immune-responsive” manner to help facilitate inflammation following cellular damage and then activate downstream actions to help mediate the inflammatory response [2,10].

When appropriate durations of rest follow the EIMD exercise bout, the damaged tissues will recover and improve physiological resilience against subsequent bouts of EIMD as part of chronic exercise adaptations [2,3,6,15]. Muscular dysfunction, inflammation, and immune dysregulation caused by EIMD will typically subside after a few days; however, the reduced muscular strength and local leukocyte accumulation have been observed up to 7 days following severe eccentric exercise events [1,4]. While sufficient rest and recovery will improve muscular resilience against future damaging bouts, HIE with minimal durations of rest are common in athletic competitions that facilitate persistent inflammation and immune dysregulation [3]. This warrants the need to investigate strategies that may mitigate inflammation and immune dysregulation often associated with EIMD and intense exercise during periods of reduced rest that can facilitate return to play.

Recently, there has been growing interest in fucoidans, fucose-rich sulfated polysaccharides, derived from either brown seaweed or echinoderms (i.e. sea cucumbers), due to its demonstrated anti-inflammatory and pro-immune responses in animal models and clinical populations [16–18]. A 4-week daily oral administration of fucoidan from *Undaria pinnatifida* (UPF) prior to an annual influenza vaccination resulted in an improved and sustained antibody response in elderly men and women for up to 20 weeks following vaccination, demonstrating fucoidans may have the potential to modulate the immune system in various situations [19]. A 4-week daily oral administration of fucoidan (*Cladosiphon novae-caledoniae*) improved chronic inflammatory biomarkers in cancer patients including reductions in systemic IL-1β and IL-6 concentrations and improved quality of life [20]. A study investigating a 4-week daily oral administration of 100 mg per day and 1000 mg per day of fucoidan (*Fucus vesiculosis*, *Macrocystis pyrifera*, *Laminaria japonica*) in healthy adults found both doses reduced basal IL-6 levels and cytotoxic T-cell activity [21]. Despite methodological differences, fucoidans have consistently been shown to reduce inflammatory markers and improve resilience of the immune system with no known toxicity or adverse side effects [16,17,21–23]. Currently, most available research involving fucoidans in humans measure alterations in basal levels of cytokines and immune cells where no inflammatory stimulus is provided. Therefore, more research is needed to elucidate whether fucoidans may influence human cytokine and immune cell response following an acute inflammatory stimulus, such as exercise in healthy populations.

Additionally, fucoidans have been implicated in anti-fatigue actions in mouse musculature during a swim-to-exhaustion task with those given fucoidan (*Laminaria japonica*) for 21 days [24]. A later study observed increased treadmill distance and an upregulation of genes to stimulate angiogenesis and mitochondrial biogenesis in mice given UPF, fucoxanthin, hesperetin, and caffeic acid for 8 weeks compared to control [25]. Recently, McBean et al. observed a significant association between muscle fiber hypertrophy and muscle force production in mice supplemented with 400 mg/kg day of a blended fucoidan from UPF and *Fucus vesiculosus* along with neutral carbohydrates via oral gavage for 4 weeks compared to control mice that received water via oral gavage, suggesting that fucoidan supplementation may improve these outcomes during exercise [26]. Research performed on UPF combined with exercise in humans is scarce. However, one pilot study indicated increased fecal lysosomes, which are antimicrobial and anti-inflammatory in action, in high-performance athletes following a 1-week daily dosage of fucoidan from *F. vesiculosus* and UPF during pre-season training camp [27]. Generally, pre-season training increases exercise intensity and volume to prepare for competition. Athletes had 73% lower levels of fecal lysosomes at baseline compared to the healthy adult comparator group, but within participant analysis following fucoidan supplementation showed athlete lysosomes count improved 43% compared to their baseline levels [27].

Fucoidans may be able to improve inflammation and immune responses following exercise. If efficacious, acute supplementation with fucoidan may lessen physiological disruption following HIE, which may benefit individuals who regularly engage in activities involving repeated efforts. Therefore, the purpose of this study was to investigate the safety and efficacy of UPF on markers of inflammation and immune response following an acute bout of HIE. The primary hypothesis was that UPF supplementation would reduce the inflammatory cytokine and immune cell response following interval-based HIE compared to placebo. Since fucoidan may attenuate levels of pro-inflammatory cytokines that have been linked to increased fatigue during exercise [2,14,28], a secondary hypothesis was that UPF supplementation may allow participants to maintain higher power output and performance during the interval-based HIE by reducing muscle fatigue.

## 2. Materials and methods

### 2.1. Participants

An equal number of healthy, adult males and females (*N* = 16; *M* = 8, *F* = 8) (Table 1) participated in supplementation and exercise testing for a total of 35 ± 1 days. To be eligible to participate, individuals were physically active, which was defined as consistent with the cumulative weekly recommended physical activity guidelines for Americans (150 –500 min per week). Professional athletes, collegiate athletes, competitive bodybuilders, or any individual that would have been competing at the elite category within their sport were not included. Females were excluded if they were pregnant, lactating, or planning on becoming pregnant during the course of the study. Participants were excluded if they had any current musculoskeletal injuries that would prevent them from completing the exercise protocol or if they had a history of any clinically significant cardiovascular, respiratory, renal, cerebrovascular, hematological, pulmonary, gastrointestinal, autoimmune, lymphatic, hepatobiliary, neurological, psychiatric, metabolic, or endocrine disorders including type 1 and type 2 diabetes. Participants were screened to exclude blood thinning medications or supplement use or for any known sensitivity or allergy for the current study products. This study was approved by the University of South Carolina Institutional Review Board, and informed consent was obtained for all participants (Pro00114427). This study was registered on ClinicalTrials.gov (NCT05181410).

**Table 1.** Participant descriptive data.

	Female (*n* = 8)	Male (*n* = 8)
Age (years)	21.7 ± 3.5	20.7 ± 1.8
Height (cm)	163.1 ± 6.8	181.1 ± 6.4
Body mass (kg)	58.8 ± 9.1	83.0 ± 11.9
Body fat (%)	24.2 ± 4.3	13.0 ± 7.7

Data are presented as mean ± standard deviation. cm=centimeter, kg=kilograms.

### 2.2. Study design and supplementation protocol

**Figure 1.**
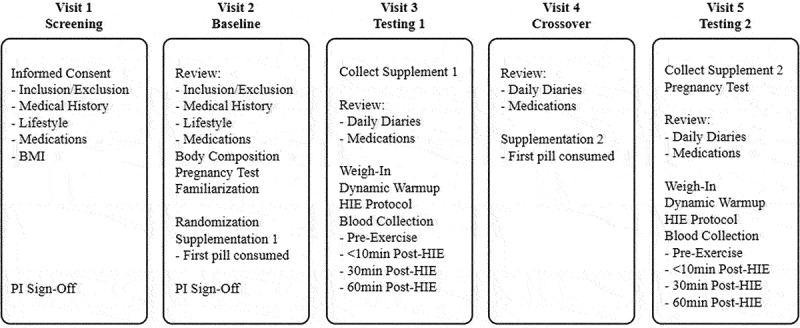
Study design: visits.

The randomized double-blind, placebo-controlled, counterbalanced crossover design of the study consisted of five visits (see Figure 1). The investigational product was an UPF fucoidan extract produced by Marinova (Cambridge, Tasmania, Australia) in the form of 500 mg capsules and the placebo (PL) was 500 mg microcrystalline cellulose capsules.

Each box represents the schedule of events for each of the five study visits.

Following a screening visit to determine eligibility, participants completed a baseline familiarization session and were subsequently stratified by sex and randomized into either of the two groups: UPF for S1 and PL for S2 (UPF/PL) or PL for S1 and UPF for S2 (PL/UPF). Participants were expected to consume one capsule in the morning and one capsule in the evening with food during supplementation periods. If a dose was accidentally missed, participants were instructed “to consume the missed dose as soon as possible” or “to consume two doses at their next regularly scheduled dosing.” Participants consumed their first dose in front of a lab research member and were monitored for 15 min for any potential adverse events (i.e. allergies), and they consumed their final dose the mornings of their testing sessions (T1/T2) to prevent any premature washout of either supplement prior to the testing sessions.

**Figure 2.**
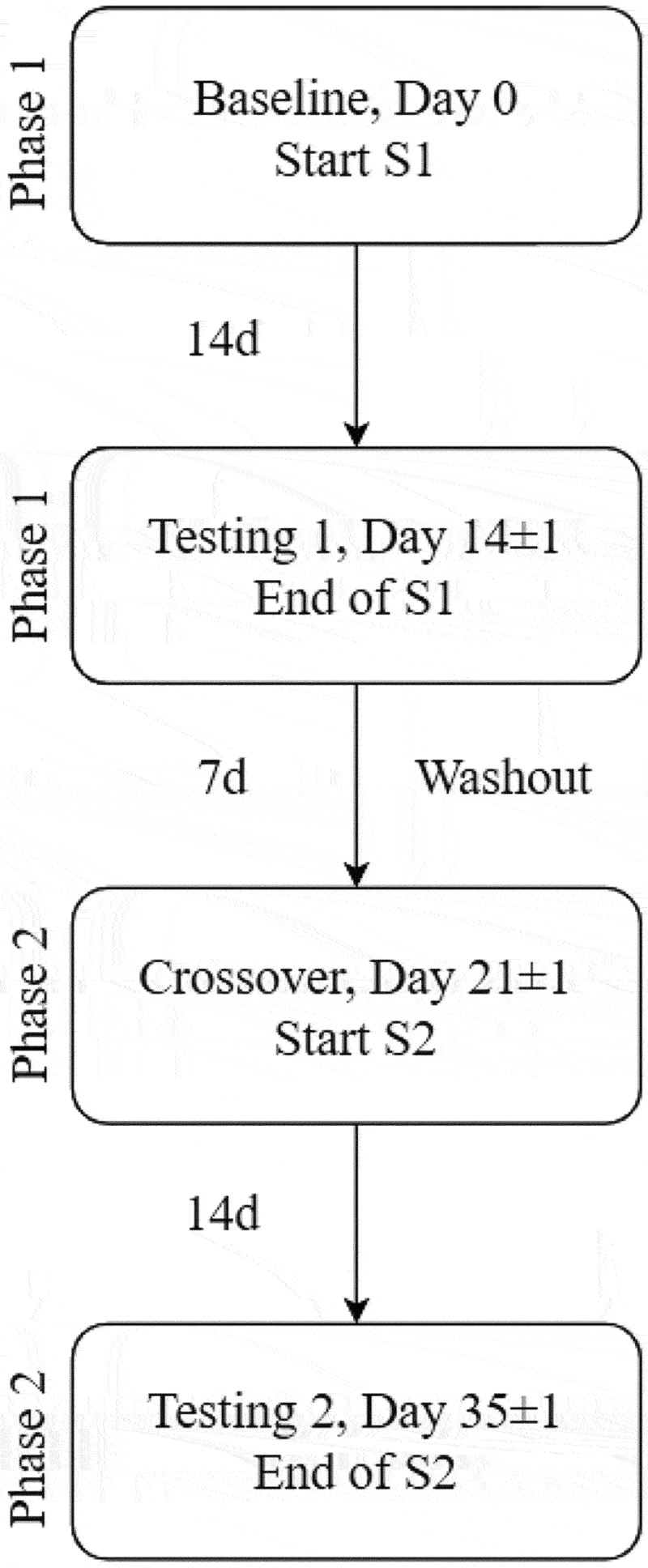
Supplementation flow.

The 2-week supplementation period was followed by the HIE testing session (T1). After the first HIE testing session, a 1-week washout phase occurred where no supplement was consumed. Throughout the interim and washout periods, participants were instructed to not alter physical activity levels or diet, and daily supplement consumption was monitored via the online electronic data collection software Medrio (San Francisco, CA, USA). After this, participants attended a crossover visit to receive the first capsule of the second supplement (S2). This was followed by 2 weeks of S2 supplementation that concluded with the second HIE testing session (T2). There was ±1 day allowed for each testing phase to accommodate participant schedules. In addition to each study visit, participants were expected to complete a daily Supplement and Health Diary (SHD) that collected routine times of supplement consumption and monitored any supplement or health changes throughout the duration of the study. SHDs were reviewed daily for completion (see Figure 2).

This diagram represents the time period of supplementation and washout as it relates to performance testing.

### 2.3. Testing sessions

At the baseline familiarization visit, participants were asked to attend the lab euhydrated and given instructions on adequate fluid consumption along with methods to remain properly hydrated. Participants were also asked to arrive 2 h fasted, to not have exercised within 12 h and to not have consumed any alcohol, caffeine, or nicotine within 12 h prior to the baseline visit. Height and initial weight were obtained on a stadiometer and calibrated scale (Health-o-meter Professional, Pelstar LLC, Alsip, IL, USA) and body composition assessment was performed via air displacement plethysmography (BodPod, Cosmed, Rome, Italy) according to the manufacturer’s guidelines. Female participants underwent a urine pregnancy test (First Response Pregnancy Test, Church & Dwight, Ewing Township, NJ, USA) to confirm a negative pregnancy status. This was done as pregnancy may have influenced both performance testing and blood biomarker results. Finally, participants underwent a brief familiarization session with the cycle ergometer (Velotron Racer-Mate, Seattle, WA, USA) that was used for both testing sessions and provided a detailed description of the HIE protocol and expectations.

Participants’ T1 and T2 testing sessions were performed at the same time of day (<1 h difference) to help control for individual diurnal variations in performance measures or biomarkers. In addition to baseline familiarization session instruction, participants were required to consume a meal 2–4 h prior to the testing session but did not consume anything else except water within 2 h prior to the testing session. Then, 24 h dietary food logs were collected for food intake the day prior to T1. These food logs were sent to the participants prior to T2, and participants were asked to replicate the diet as closely as possible, which was verified by the administration of a second 24 h food log prior to T2. A second pregnancy test was administered to female participants at T2 before the initiation of blood draws and the exercise protocol to verify a negative pregnancy status for the duration of the study. Same-day participant body mass was obtained prior to HIE protocol to update and program the equipment software (Velotron, Wingate version 1.0.2). Following completion, the first blood draw was obtained, and the participant initiated the HIE protocol.

### 2.4. High-intensity exercise protocol

All participants underwent a standardized 7 min dynamic warm-up then pedaled the cycle ergometer for 5 min using a self-selected cadence at 75 W. Participants then completed a 3 min rest while seated on the cycle ergometer. The protocol for the Wingate Anaerobic Tests (WAnT) was derived from previous research with resistance set at 0.10 kP/kg body mass [29]. The testing protocol included one 30 s WAnT followed by a 5 min rest, then eight 10 s WAnT intervals interspersed with 2 min rest periods. This HIE protocol was used to determine mean power (MP) and peak power (PP) during the 30 s WAnT, and when averaged over the total exercise time (30 s WAnT +8, 10 s WAnTs).

### 2.5. Blood collection

Blood was collected at four timepoints on T1 and T2: prior to the initiation of any exercise (timepoint A), within 10 min following HIE (timepoint B), 30 min post-exercise (timepoint C), and 60 min post-exercise (timepoint D). The first blood draws occurred following a 10 min rest in a seated position. A venous catheter (Becton, Dickinson, and Company, Franklin Lakes, NJ, USA) was inserted into the antecubital vein and was used for all four blood draws. One participant (female) was excluded from blood analysis due to inability to retrieve sufficient blood from her veins; however, she was included in HIE performance analysis.

Approximately 25 mL of blood was drawn at every timepoint for approximately 100 mL of blood drawn per testing session using a serum separator tube (SST) and two dipotassium ethylenediaminetetraacetic acid tubes (K2EDTA; Becton, Dickinson & Co., Franklin Lakes, NJ, USA). The K2EDTA tubes were sent to a CLIA-certified laboratory (Bio-Reference Laboratories, Inc.; Elmwood Park, NJ, USA) for whole blood and immune system analysis within 24 h of collection. Blood composition analysis included total white blood cell count (WBC), red blood cell count (RBC), hemoglobin (HGB), hematocrit (HCT), mean corpuscular volume (MCV), mean corpuscular hemoglobin (MCH), MCH concentration (MCHC), RBC distribution width (RDW), platelet count, and mean platelet volume (MPV). HGB and HCT were used as a standard to calculate plasma value shifts with acute exercise and therefore are not presented in the results. The immune marker panel included leukocyte concentrations of neutrophils, lymphocytes, monocytes, eosinophils, basophils, natural killer cells (CD16 + CD56) (NKC), T-lymphocytes (CD3), and B- lymphocytes (CD19). The panel also included the concentrations of T-helper cells, which present CD4 glycoproteins (CD4 cells), and *T*-suppressor cells, which present CD8 glycoproteins (CD8 cells).

SST vials were allowed to stand for 30 min following collection and inversion, prior to processing where vials were centrifuged for 15 min at 3300×*g*. Serum was then pipetted into aliquot tubes and stored at –80°C until cytokine analysis of IL-1β, IL-6, and IL-10. Cytokine analysis was performed following the collection of all samples using the commercially available magnetic-bead assay kits (Human TH17 Multiplex Assay, EMD Millipore Corporation, Burlington, MA, USA) and a magnetic multiplex analyzer (MAGPIX, Luminex, Austin, TX, USA) with the coefficient of variation (CV) between 6.7% and 8.4%. Blood biomarkers were adjusted to account for plasma volume shifts during exercise using the hemoglobin and hematocrit adjustment factor method as described in Greenleaf et al. [30].

### 2.6. Statistical analysis

Linear mixed-effects models in a 2 (condition) × 4 (time) design were used to assess the outcome variables; the models included product sequence (covariate), condition, time, and time-by- condition as fixed factors and subject ID as a random intercept, which allows the model intercept to vary based on the subject. Given the within-subject study design and the non-independent nature of the values of each outcome variable, the application of such linear mixed-effects models and the inclusion of a by-subject random intercept is appropriate [31]. To determine the effects of sex, additional linear mixed-effects models were conducted to test for main effects of sex, sex-by-condition interactions, sex-by-time interactions, and three-way sex-by-condition-by-time interactions. Post hoc analyses were run when the main effect or interaction effect reached significance using the Dunnett adjustment method. Additional outcomes, including average peak power (PP) and average mean power (MP), were assessed between conditions for the 30 s WAnT and for total exercise duration (30 s WAnT +8, 10 s WAnT intervals [Total PP and Total MP]) via a mixed-model approach. Effect size was calculated with Cohen’s *d* (small = 0.2; medium = 0.5; large = 0.8). All effect sizes (*d*) are compared to timepoint A. Additional effect sizes were calculated in the case of significant interactions in order to compare the effects of conditions within time. Significance was set at α = .05. Statistical analysis was performed using the statistical software R (version 4.2.0).

## 3. Results

One male participant was excluded from exercise and blood analysis due to low supplementation compliance (73.1%). One female participant was excluded from blood analysis due to difficulty in obtaining sufficient blood, but she was included in the exercise analysis. Excluding the low compliance participant, average supplement compliance was 97.5 ± 5.3% and SHD completion was 100%. Data are presented in Table 1.

### 3.1. Complete blood count

No significant time-by-condition interactions or main effect of condition were observed in complete blood count markers: WBC, RBC, MCV, MCH, MCHC, RDW, platelets, or MPV (*p <* .05). A significant time effect was observed for WBC, RBC, platelet count, and MPV (*p < *.05). Post hoc analysis revealed greater values at timepoint B than timepoint A for WBC (*p* < .0001), RBC (*p* < 0.0001), platelet count (p < .0001), and MPV (*p* < .001) for both supplementation conditions. No time main effects were observed for MCV, MCH, MCHC, or RDW (*p* >  .05). Data are presented in Table 2.

**Table 2.** Complete blood count analysis following high-intensity exercise.

			PL	*d*	UPF	*d*
WBC (1000/µL)	A	Overall	6.5 ± 1.6	–	6.4 ± 1.3	–
		Female	7.2 ± 1.9	–	6.6 ± 1.2	–
		Male	5.7 ± 0.8	–	6.2 ± 1.5	–
	B^#^	Overall	12.1 ± 3.5	3.50	11.7 ± 2.8	3.31
		Female	12.4 ± 3.2	2.74	11.4 ± 2.9	4.00
		Male	11.7 ± 3.9	7.50	11.9 ± 2.9	3.80
	C	Overall	6.9 ± 1.8	0.25	7.2 ± 1.9	0.50
		Female	7.3 ± 1.9	0.05	7.5 ± 1.6	0.75
		Male	6.6 ± 1.9	1.13	7.0 ± 2.1	0.53
	D	Overall	7.3 ± 2.6	0.50	7.3 ± 2.3	0.56
		Female	8.5 ± 3.2	0.68	8.7 ± 2.4	1.75
		Male	6.4 ± 1.5	0.88	6.0 ± 1.4	-0.13
RBC (1,000,000/µL)	A	Overall	4.8 ± 0.4	–	4.6 ± 0.5	–
		Female	4.5 ± 0.3	–	4.3 ± 0.6	–
		Male	5.1 ± 0.3	–	5.0 ± 0.2	–
	B^#^	Overall	5.2 ± 0.7	1.00	5.2 ± 0.8	1.20
		Female	4.7 ± 0.4	0.67	4.7 ± 0.8	0.67
		Male	5.8 ± 0.6	2.33	5.8 ± 0.5	4.00
	C	Overall	4.7 ± 1.0	–0.25	5.1 ± 0.9	1.00
		Female	4.1 ± 0.8	–1.33	4.3 ± 0.4	0.00
		Male	5.1 ± 0.9	0.00	5.7 ± 0.6	3.50
	D	Overall	4.6 ± 0.9	–0.50	4.6 ± 0.7	0.00
		Female	3.9 ± 0.9	–2.00	4.1 ± 0.7	–0.33
		Male	5.1 ± 0.3	0.00	5.0 ± 0.4	0.00
MCV (fL)	A	Overall	90.0 ± 2.6	–	90.0 ± 2.8	–
		Female	90.9 ± 3.5	–	90.6 ± 3.8	–
		Male	89.1 ± 0.9	–	89.4 ± 1.5	–
	B	Overall	94.9 ± 8.2	1.88	94.4 ± 13.2	1.57
		Female	93.1 ± 9.7	0.63	94.1 ± 17.9	0.92
		Male	96.7 ± 6.7	8.44	94.8 ± 7.5	3.60
	C	Overall	88.7 ± 14.7	–0.50	96.4 ± 9.6	2.29
		Female	87.6 ± 15.9	–0.95	92.6 ± 7.5	0.53
		Male	89.7 ± 14.8	0.67	99.7 ± 10.4	6.87
	D	Overall	88.9 ± 8.5	–0.42	89.8 ± 7.6	–0.07
		Female	85.9 ± 10.9	–1.43	89.1 ± 11.2	–0.39
		Male	91.5 ± 5.5	2.67	90.4 ± 3.3	0.67
MCH (pg)	A	Overall	30.0 ± 1.2	–	30.1 ± 1.2	–
		Female	29.9 ± 1.6	–	30.2 ± 1.6	–
		Male	30.1 ± 0.8	–	30.1 ± 0.8	–
	B	Overall	31.6 ± 2.7	1.33	31.1 ± 4.5	0.83
		Female	31.0 ± 3.4	0.69	30.9 ± 6.0	0.44
		Male	32.3 ± 1.7	2.75	31.2 ± 2.8	1.38
	C	Overall	29.6 ± 4.8	–0.33	32.0 ± 2.9	1.58
		Female	28.9 ± 5.1	-0.63	30.6 ± 2.4	0.25
		Male	30.1 ± 4.8	0.00	33.3 ± 2.9	4.00
	D	Overall	29.7 ± 3.1	–0.25	29.8 ± 2.5	–0.25
		Female	28.2 ± 3.7	–1.06	29.4 ± 3.5	–0.50
		Male	31.0 ± 1.9	1.13	30.2 ± 1.5	0.13
MCHC (g/dL)	A	Overall	33.3 ± 0.8	–	33.5 ± 0.7	–
		Female	32.9 ± 0.7	–	33.3 ± 0.5	–
		Male	33.8 ± 0.7	–	33.6 ± 0.9	–
	B	Overall	34.8 ± 2.7	1.87	34.0 ± 4.4	0.71
		Female	33.9 ± 3.2	1.43	33.4 ± 5.5	–0.20
		Male	35.7 ± 2.0	2.71	34.6 ± 3.4	1.11
	C	Overall	32.8 ± 5.2	–0.63	35.4 ± 3.1	2.71
		Female	31.8 ± 5.3	–1.57	33.3 ± 2.1	–0.40
		Male	33.6 ± 5.3	–0.29	37.2 ± 2.7	4.00
	D	Overall	33.1 ± 3.9	–0.25	32.9 ± 2.9	–0.86
		Female	31.1 ± 4.7	–2.57	32.0 ± 3.7	–3.00
		Male	34.8 ± 2.1	1.43	33.6 ± 1.9	0.00
RDW (%)	A	Overall	12.8 ± 0.7	–	12.7 ± 0.6	–
		Female	12.9 ± 0.8	–	12.6 ± 0.5	–
		Male	12.7 ± 0.7	–	12.9 ± 0.7	–
	B^#^	Overall	12.8 ± 0.7	–0.00	12.8 ± 1.2	0.17
		Female	12.8 ± 0.7	–0.13	12.4 ± 0.6	–0.40
		Male	12.8 ± 0.7	0.14	13.2 ± 1.6	0.43
	C	Overall	12.7 ± 0.6	–0.14	12.7 ± 0.6	0.00
		Female	12.7 ± 0.6	–0.25	12.4 ± 0.5	–0.40
		Male	12.8 ± 0.6	0.14	13.0 ± 0.7	0.14
	D	Overall	12.8 ± 0.7	0.00	12.7 ± 0.6	0.00
		Female	12.9 ± 0.8	0.00	12.4 ± 0.4	–0.40
		Male	12.8 ± 0.7	0.14	13.0 ± 0.7	0.14
Platelets (1000/µL)	A	Overall	259.7 ± 64	–	248.5 ± 40.5	–
		Female	285.9 ± 69.5	–	256.4 ± 47.2	–
		Male	233.6 ± 49.5	–	240.6 ± 34.4	–
	B^#^	Overall	370.5 ± 115.4	1.73	352.4 ± 109.7	2.57
		Female	396.7 ± 143.3	1.59	365.3 ± 146.2	2.31
		Male	344.3 ± 82.1	0.34	339.4 ± 65.6	2.87
	C	Overall	254.4 ± 60.0	–0.08	261.9 ± 53.1	0.33
		Female	258.8 ± 66.4	–0.39	252.4 ± 48.2	–0.08
		Male	250.6 ± 59.0	0.30	270.1 ± 59.4	0.86
	D	Overall	245.6 ± 102.3	–0.22	239.7 ± 91.2	–0.22
		Female	249.0 ± 142.9	–0.53	255.9 ± 128.4	–0.01
		Male	242.7 ± 62.3	0.18	225.8 ± 49.3	–0.43
MPV (fL)	A	Overall	10.7 ± 0.9	–	10.8 ± 0.6	–
		Female	10.5 ± 0.8	–	10.7 ± 0.5	–
		Male	10.9 ± 0.9	–	11.0 ± 0.7	–
	B^#^	Overall	11.6 ± 1.2	1.00	11.5 ± 1.7	1.16
		Female	11.1 ± 0.9	0.75	11.3 ± 2.0	1.20
		Male	12.0 ± 1.3	1.22	11.7 ± 1.3	1.00
	C	Overall	10.8 ± 2.0	0.11	11.8 ± 1.2	1.67
		Female	10.6 ± 2.2	0.13	11.3 ± 0.6	1.20
		Male	11.0 ± 1.9	0.11	12.1 ± 1.5	1.57
	D	Overall	10.8 ± 1.0	0.11	10.8 ± 0.8	0.00
		Female	10.2 ± 1.1	–0.38	10.6 ± 1.0	–0.20
		Male	11.2 ± 0.8	0.33	10.9 ± 0.7	–0.14

Data are presented as adjusted mean ± standard deviation. All effect sizes (*d*) are compared to timepoint A. # indicates a difference from timepoint A (*p < 0*.05).

PL = placebo, UPF = *Undaria pinnatifida* fucoidan, WBC = white blood cells, RBC = red blood cells.

MCV = mean corpuscular volume, MCH = mean corpuscular hemoglobin, MCHC = mean corpuscular hemoglobin concentration, RDW = RBC distribution width, MPV = mean platelet volume, µL = microliter, pg = picogram, g/dL = grams per deciliter, fL= femtoliter.

### 3.2. Complete blood count sex comparison

No sex interactions or main effects of sex were found for WBC, MCV, MCH, RDW, platelets, or MPV (*p* > .05). Main effect of sex was found for RBC (*p* = .0002) and MCHC (*p* = .03). Post hoc analysis indicated that males experienced higher RBC (*p* = .0002) and MCHC (*p* = .03) concentrations than females. Data are presented in Table 2.

### 3.3. Immune system response

No significant time-by-condition interactions or main effect of condition were found for immune markers: neutrophils, lymphocytes monocytes, eosinophils, basophils, NKC, T-lymphocytes, and B-lymphocytes (*p* > .05). A significant time effect was observed for neutrophils, lymphocytes, monocytes, eosinophils, basophils, NKC, T-lymphocytes, B-lymphocytes (*p* < .05). Post hoc analysis revealed timepoint B had higher values than timepoint A for neutrophils (*p* < .0001), lymphocytes (*p* < .0001), monocytes (*p* < .001), eosinophils (*p* < .001), basophils (*p* < .0001), NKC (*p* < .001), T-lymphocytes (*p* < .0001), and B-lymphocytes (*p* < .001) for both conditions. Post hoc analysis also revealed lower values at timepoint D than timepoint A for concentrations of lymphocytes (*p* = .017), NKC (*p* = .03), T-lymphocytes (*p* = .03), and B-lymphocytes (*p* = .002). Additionally, post hoc analysis revealed that the concentration of neutrophils was greater at timepoint C (*p* = .03) and D (*p* < .0001) than at timepoint A. Data are presented in Table 3.

**Table 3.** Immune system analysis following high-intensity exercise.

			PL	*d*	UPF	*d*
Neutrophils (1000/µL)	A	Overall	3.6 ± 1.2	–	3.6 ± 1.0	–
		Female	4.0 ± 1.6	–	3.7 ± 0.9	–
		Male	3.1 ± 0.5	–	3.6 ± 1.1	–
	B^#^	Overall	5.7 ± 2.0	1.75	5.7 ± 1.7	2.10
		Female	5.9 ± 2.4	1.19	5.7 ± 1.8	2.22
		Male	5.4 ± 1.7	4.60	5.7 ± 1.7	1.91
	C^#^	Overall	4.2 ± 1.4	0.50	4.4 ± 1.3	0.80
		Female	4.7 ± 1.8	0.44	4.7 ± 1.3	1.11
		Male	3.8 ± 1.0	1.40	4.1 ± 1.3	0.45
	D^#^	Overall	5.1 ± 2.1	1.25	5.0 ± 2.1	1.40
		Female	6.1 ± 2.5	1.31	6.2 ± 2.3	2.78
		Male	4.2 ± 1.2	2.20	3.9 ± 1.1	0.27
Lymphocytes (1000/µL)	A	Overall	2.2 ± 0.8	–	2.1 ± 0.5	–
		Female	2.5 ± 0.8	–	2.1 ± 0.5	–
		Male	2.0 ± 0.8	–	2.0 ± 0.5	–
	B^#^	Overall	5.2 ± 2.2	3.75	4.9 ± 1.4	5.60
		Female	5.3 ± 1.3	3.50	4.9 ± 1.3	5.60
		Male	5.1 ± 2.9	3.88	5.0 ± 1.5	6.00
	C	Overall	2.0 ± 0.9	–0.25	2.1 ± 0.8	0.00
		Female	1.8 ± 0.3	–0.88	2.0 ± 0.6	–0.20
		Male	2.1 ± 1.2	0.13	2.2 ± 0.9	0.40
	D^#^	Overall	1.6 ± 0.7	–0.75	1.6 ± 0.5	–1.00
		Female	1.7 ± 0.7	–1.00	1.8 ± 0.6	–0.60
		Male	1.6 ± 0.7	–0.50	1.5 ± 0.5	–1.00
Monocytes (1000/µL)	A	Overall	0.5 ± 0.1	–	0.5 ± 0.1	–
		Female	0.5 ± 0.1	–	0.4 ± 0.09	–
		Male	0.5 ± 0.08	–	0.6 ± 0.1	–
	B^#^	Overall	0.9 ± 0.2	4.00	0.9 ± 0.2	4.00
		Female	0.8 ± 0.2	3.00	0.8 ± 0.2	2.00
		Male	0.9 ± 0.2	4.00	0.9 ± 0.2	5.00
	C	Overall	0.5 ± 0.1	0.00	0.5 ± 0.1	0.00
		Female	0.5 ± 0.2	0.00	0.5 ± 0.1	–1.00
		Male	0.5 ± 0.1	1.25	0.5 ± 0.2	1.00
	D	Overall	0.5 ± 0.1	0.00	0.5 ± 0.1	0.00
		Female	0.5 ± 0.1	0.00	0.5 ± 0.1	–1.00
		Male	0.5 ± 0.1	0.00	0.4 ± 0.1	0.00
Eosinophils (1000/µL)	A	Overall	0.1 ± 0.1	–	0.1 ± 0.1	–
		Female	0.1 ± 0.1	–	0.1 ± 0.0	–
		Male	0.1 ± 0.1	–	0.1 ± 0.1	–
	B^#^	Overall	0.2 ± 0.1	1.11	0.2 ± 0.1	1.11
		Female	0.2 ± 0.1	1.11	0.2 ± 0.1	1.11
		Male	0.2 ± 0.1	1.11	0.2 ± 0.1	1.11
	C	Overall	0.1 ± 0.1	0.00	0.1 ± 0.1	0.00
		Female	0.1 ± 0.1	0.00	0.1 ± 0.1	0.00
		Male	0.1 ± 0.1	0.00	0.1 ± 0.1	0.00
	D	Overall	0.1 ± 0.1	0.00	0.1 ± 0.1	0.00
		Female	0.1 ± 0.08	0.00	0.1 ± 0.07	0.00
		Male	0.1 ± 0.1	0.00	0.07 ± 0.06	0.00
Basophils (1000/µL)	A	Overall	0.05 ± 0.02	–	0.04 ± 0.01	–
		Female	0.06 ± 0.02	–	0.04 ± 0.01	–
		Male	0.04 ± 0.01	–	0.04 ± 0.02	–
	B^#^	Overall	0.08 ± 0.02	1.50	0.08 ± 0.02	4.00
		Female	0.09 ± 0.02	1.50	0.08 ± 0.03	4.00
		Male	0.08 ± 0.02	4.00	0.07 ± 0.03	1.50
	C	Overall	0.05 ± 0.02	0.00	0.05 ± 0.02	1.00
		Female	0.06 ± 0.02	0.00	0.05 ± 0.01	1.00
		Male	0.05 ± 0.02	1.00	0.04 ± 0.02	0.00
	D	Overall	0.04 ± 0.02	-0.50	0.04 ± 0.02	0.00
		Female	0.04 ± 0.02	–1.00	0.05 ± 0.02	1.00
		Male	0.04 ± 0.02	0.00	0.04 ± 0.02	0.00
NKC (cells/µL)	A	Overall	321.9 ± 165.3	–	285.6 ± 96.0	–
		Female	392.4 ± 189.5	–	306.0 ± 81.8	–
		Male	251.3 ± 108.1	–	265.1 ± 110.9	–
	B^#^	Overall	1477.7 ± 539.6	6.99	1436.6 ± 478.0	11.99
		Female	1629.2 ± 447.1	6.53	1506.8 ± 644.3	14.81
		Male	1326.3 ± 614.3	9.94	1376.3 ± 319.2	10.02
	C	Overall	255.0 ± 139.9	–0.40	245.1 ± 90.3	–0.42
		Female	220.2 ± 31.6	–0.91	226.7 ± 39.8	–0.98
		Male	284.9 ± 189.9	0.31	260.9 ± 119.8	–0.04
	D^#^	Overall	132.1 ± 70.8	–1.15	121.6 ± 38.2	–1.71
		Female	135.6 ± 58.3	–1.36	125.0 ± 34.5	–2.23
		Male	129.2 ± 84.7	–1.13	118.6 ± 43.5	–1.32
T-Lymphocytes (cells/µL)	A	Overall	1589.9 ± 546.9	–	1475.4 ± 417.9	–
		Female	1722.9 ± 538.1	–	1510.3 ± 457.6	–
		Male	1457.0 ± 563.2	–	1440.4 ± 407.5	–
	B^#^	Overall	3144.3 ± 1562.9	2.84	2949.1 ± 897	3.53
		Female	3044.5 ± 987.0	2.46	2781.3 ± 705.1	2.78
		Male	3244.0 ± 2072.5	3.17	3092.9 ± 1069	4.06
	C	Overall	1442.5 ± 669.4	–0.27	1570.0 ± 596.9	0.23
		Female	1320.4 ± 294.1	–0.75	1499.6 ± 527.0	–0.02
		Male	1547.2 ± 892.5	0.16	1630.4 ± 687.1	0.47
	D^#^	Overall	1216.3 ± 533.2	–0.68	1144.1 ± 500.6	–0.79
		Female	1232.7 ± 567.5	–0.91	1353.0 ± 456.4	–0.34
		Male	1202.2 ± 547.5	–0.45	965.0 ± 496.6	–1.17
B-Lymphocytes (cells/µL)	A	Overall	297.3 ± 137.0	–	276.3 ± 135.3	–
		Female	311.3 ± 95.3	–	282.6 ± 123.1	–
		Male	283.3 ± 176.5	–	270.0 ± 156.3	–
	B^#^	Overall	513.1 ± 252.8	1.58	476.8 ± 211.0	1.48
		Female	541.0 ± 214.3	2.41	492.5 ± 210.1	1.71
		Male	485.2 ± 301.3	1.14	463.4 ± 227.6	1.24
	C	Overall	249.3 ± 119.6	–0.35	259.6 ± 133.1	–0.12
		Female	253.0 ± 84.5	–0.61	260.8 ± 119.6	–0.18
		Male	246.1 ± 150.5	–0.21	258.6 ± 153.4	–0.07
	D^#^	Overall	231.9 ± 119.3	–0.48	223.7 ± 130.2	–0.39
		Female	256.9 ± 124.9	–0.57	239.5 ± 150.2	–0.35
		Male	210.5 ± 119.6	–0.41	210.1 ± 121.1	–0.38

Data are presented as adjusted mean ± standard deviation. All effect sizes (*d*) are compared to timepoint A. # indicates a difference from timepoint A (*p* < .05).

PL = placebo, UPF = *Undaria pinnatifida* fucoidan, µL = microliter.

### 3.4. Immune system response sex comparison

A sex-by-time interaction was found for neutrophils (*p = *.03). Post-hoc analysis revealed females experienced higher concentrations at timepoint B (*p* < .001) and D (*p < *.001) compared to A, whereas males experienced greater concentrations of neutrophils at timepoint B compared to timepoint A only (*p* <. 001). No sex interactions or main effects of sex were found for lymphocytes, monocytes, eosinophils, basophils, NKC, T-lymphocytes, or B-lymphocytes (*p* > .05). Data are presented in Table 3.

### 3.5. CD4 and CD8 response

No significant time-by-condition interactions or main effect of condition were found for CD4 or CD8 biomarkers (*p* > .05). A significant time effect was observed for CD4 and CD8 (*p* < .05). Post hoc analysis revealed timepoint B had higher values than timepoint A for CD4 (*p* < .0001) and CD8 (*p* < .001) markers for both conditions. Post hoc analysis also revealed lower values at timepoint D than timepoint A for concentrations of CD4 (*p*=.01). Data are presented in Table 4.

**Table 4.** CD4 and CD8 concentrations following high-intensity exercise.

			PL	*d*	UPF	*d*
CD4 (cells/µL)	A	Overall	850.9 ± 293.2	–	804.0 ± 244.7	–
		Female	978.6 ± 300.5	–	864.7 ± 273.7	–
		Male	723.1 ± 240.7	–	743.3 ± 215.1	–
	B^#^	Overall	1345.8 ± 510.5	1.69	1323.3 ± 376.9	2.12
		Female	1434.7 ± 514.3	1.52	1375.9 ± 440.1	1.87
		Male	1257.0 ± 530.8	2.22	1278.2 ± 342.8	2.49
	C	Overall	722.7 ± 187.1	–0.44	819.8 ± 269.6	0.06
		Female	744.6 ± 175.6	–0.78	836.0 ± 306.0	–0.10
		Male	703.9 ± 208.4	–0.08	805.8 ± 258.5	0.29
	D^#^	Overall	716.9 ± 292.8	–0.46	730.8 ± 250.0	–0.30
		Female	803.4 ± 380.7	–0.58	844.7 ± 306.3	–0.07
		Male	642.7 ± 191.7	–0.33	633.3 ± 150.9	–0.51
CD8 (cells/µL)	A	Overall	656.2 ± 312.2	–	608.6 ± 210.2	–
		Female	672.3 ± 303.0	–	603.7 ± 222.7	–
		Male	640.1 ± 344.7	–	613.4 ± 214.6	–
	B^#^	Overall	1579.0 ± 1042.1	2.96	1417.9 ± 515.1	3.85
		Female	1448.4 ± 581.3	2.56	1295.5 ± 266.0	3.11
		Male	1709.5 ± 1405.4	3.10	1522.8 ± 666.3	4.24
	C	Overall	622.5 ± 419.2	–0.11	664.3 ± 309.5	0.26
		Female	508.6 ± 176.3	–0.54	617.1 ± 229.1	0.06
		Male	720.2 ± 549.1	0.23	704.8 ± 379.1	0.43
	D	Overall	455.8 ± 283.1	–0.64	471.9 ± 195.4	–0.65
		Female	404.4 ± 248.1	–0.88	485.7 ± 179.4	–0.53
		Male	499.8 ± 322.7	–0.41	460.1 ± 221.8	–0.71

Data are presented as adjusted mean ± standard deviation. All effect sizes (*d*) are compared to timepoint A. #: Indicates a difference from timepoint A (*p*<.05). PL=placebo, UPF=*undaria pinnatifida* fucoidan, µL=microliter.

### 3.6. CD4 and CD8 sex response

No sex interactions or main effects of sex were observed for CD4 or CD8 concentrations (*p >* .05). Data are presented in Table 4.

### 3.7. Cytokine response

**Figure 3.**
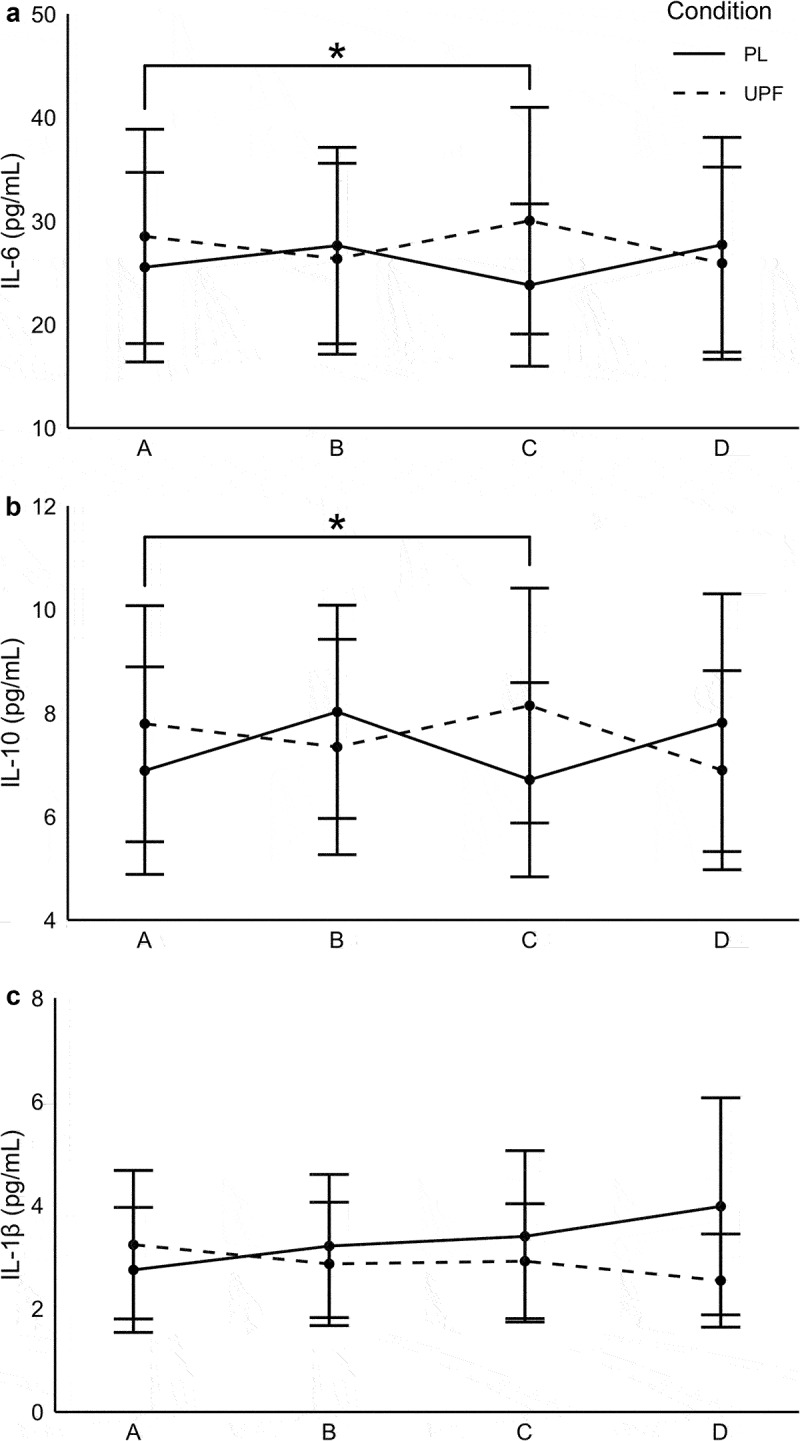
Cytokine response following high-intensity exercise.

A time-by-condition interaction was observed for IL-6 concentrations between PL and UPF (*p =* .01). Post hoc analysis revealed greater IL-6 concentrations at timepoint C for UPF compared to PL (*d*_*PLC – UPFC*_ = 0.21; *p* = .002). A time-by-condition interaction was observed for IL-10 (*p* = .008). Post hoc analysis indicated higher IL-10 concentrations for UPF at timepoint C compared to PL (*d*_*PLC – UPFC*_ = 0.20; *p* = .005). No significant differences were seen with IL-1β (*p* > .05). No main effect of condition or time was observed for IL-6, IL-10, or IL-1β (*p* > .05). Data are presented in Figure 3.

Graph A (top) represents changes in interleuken-6 concentrations pre and up to 60 min post high-intensity exercise bout with and without *Undaria pinnatifida* fucoidan supplementation.

Graph B (top) represents changes in interleukin-10 concentrations pre and up to post high-intensity exercise bout with and without *Undaria pinnatifida* fucoidan supplementation.

Graph C (top) represents changes in interleukin-1beta concentrations pre- and up to post high-intensity exercise bout with and without *Undaria pinnatifida* fucoidan supplementation.

*denotes significant difference from timepoint A in UPF.

PL = placebo, UPF = *Undaria pinnatifida* fucoidan, pg = picogram, mL = milliliter.

### 3.8. Cytokine sex comparison

No sex interactions or main effects of sex were observed for IL-6 and IL-10. A sex-by-condition interaction was observed for IL-1β (*p* = .03), with post hoc analysis revealing no differences between groups for females (*p* = .60); however, males showed lower IL-1β concentrations with the UPF supplementation condition (*p* = .03). Data are presented in Table 5.

**Table 5.** Inflammatory cytokines analysis following high-intensity exercise.

			PL	*d*	UPF	*d*
IL-6^*#^ (pg/mL)	A	Overall	25.5 ± 34.3	–	28.5 ± 37.3	–
		Females	21.0 ± 23.3	–	23.7 ± 29.4	–
		Males	30.1 ± 44.2	–	32.6 ± 45.0	–
	B	Overall	27.6 ± 35.5	0.06	26.3 ± 34.5	–0.06
		Females	22.2 ± 21.5	0.05	21.2 ± 20.4	–0.09
		Males	33.1 ± 46.9	0.07	31.5 ± 45.9	–0.02
	C	Overall	23.8 ± 29.3	–0.05	30.0 ± 41.0	0.04
		Females	19.7 ± 20.7	–0.06	23.2 ± 24.5	–0.02
		Males	27.9 ± 37.4	–0.05	36.9 ± 54.1	0.10
	D	Overall	27.7 ± 38.8	0.06	25.9 ± 34.7	–0.07
		Females	23.2 ± 27.3	0.09	19.9 ± 19.9	–0.13
		Males	32.2 ± 49.7	0.05	31.9 ± 46.2	-0.02
IL-10^*#^ (pg/mL)	A	Overall	6.9 ± 7.5	–	7.8 ± 8.2	–
		Females	6.5 ± 6.0	–	8.0 ± 7.2	–
		Males	7.3 ± 9.3	–	7.7 ± 9.6	–
	B	Overall	8.0 ± 7.4	0.15	7.3 ± 7.8	–0.06
		Females	6.7 ± 5.0	0.03	6.6 ± 4.8	–0.19
		Males	9.6 ± 9.8	0.24	8.1 ± 10.3	0.04
	C	Overall	6.7 ± 7.0	–0.03	8.1 ± 8.5	0.04
		Females	6.0 ± 4.7	–0.08	7.7 ± 6.3	–0.04
		Males	7.4 ± 9.1	0.01	8.5 ± 10.8	0.08
	D	Overall	7.8 ± 9.3	0.12	6.9 ± 7.2	–0.11
		Females	7.1 ± 6.8	0.10	6.3 ± 4.7	–0.24
		Males	8.5 ± 11.9	0.13	7.5 ± 9.4	–0.02
IL-1β (pg/mL)	A	Overall	2.7 ± 3.8	–	3.2 ± 4.3	–
		Females	1.0 ± 1.2	–	1.4 ± 1.5	–
		Males	5.4 ± 5.1	–	5.5 ± 5.8	–
	B	Overall	3.2 ± 4.4	0.13	2.9 ± 3.8	–0.07
		Females	1.0 ± 1.2	0.00	1.2 ± 1.3	–0.13
		Males	6.5 ± 5.5	0.22	5.4 ± 5.1	–0.02
	C	Overall	3.4 ± 4.7	0.18	2.9 ± 3.5	–0.07
		Females	1.3 ± 1.3	0.25	1.4 ± 1.2	0.00
		Males	5.5 ± 6.2	0.02	5.2 ± 4.7	–0.05
	D	Overall	4.0 ± 6.3	0.34	2.5 ± 2.8	–0.16
		Females	1.5 ± 1.6	0.42	1.2 ± 1.1	–0.13
		Males	7.1 ± 8.9	0.33	4.6 ± 3.6	–0.16

Data are presented as adjusted mean ± standard deviation. All effect sizes (*d*) are compared to timepoint A. # indicates a difference from timepoint A (*p < *.05). *# indicates a condition-by-time interaction (*p < *.05).

PL = placebo, UPF = *Undaria pinnatifida* fucoidan, pg = picogram, mL = milliliter.

### 3.9. Performance outcomes

Average peak power and MP did not differ between UPF and PL on either the 30 s WAnT or PP and MP for the total exercise session (*p >* .05). No sex-by-condition interactions were found (*p > *.05). Main effects of sex were found for all performance variables (*p <* .05) with post hoc analysis indicating that males produced more absolute power than females (*p < *.05). PP and MP data are shown in Table 6.

**Table 6.** High-intensity exercise peak power and mean power.

		30s PP (W)	30s MP (W)	Average PP (W)	Average MP (W)
PL	Overall	1013 ± 308	614 ± 192	1078 ± 323	684 ± 192
	Females	784 ± 128	460 ± 71	828 ± 152	537 ± 98
	Males	1274 ± 231	790 ± 111	1364 ± 195	852 ± 112
UPF	Overall	1054 ± 339	612 ± 178	1086 ± 347	690 ± 202
	Females	802 ± 167	475 ± 73	821 ± 172	533 ± 91
	Males	1341 ± 234	770 ± 117	1388 ± 216	869 ± 125

Data are presented as mean ± standard deviation, W = watts, PP = peak power, MP = mean power, s = seconds, PL = placebo, UPF = *Undaria pinnatifida* fucoidan.

## 4. Discussion

Significant changes in biomarkers occurred following HIE, indicating an overall profound effect on the inflammatory and immune system. While there were limited differences in biomarker response between supplementation conditions, a notable difference in cytokine changes occurred with UPF compared to placebo, which may have implications for recovery. Interestingly, the inflammatory and immune system responses following both supplementation conditions were generally similar between males and females (with the exception of IL-1β and neutrophils), suggesting no apparent differences between sexes. Importantly, no adverse events were reported throughout the study period, indicating a positive safety profile of UPF.

IL-6 production is proportional to exercise intensity [3,10,12] and typically peaks directly following HIE and within 1 h for MIE [15]. It immediately acts in a pro-inflammatory manner and later helps mediate the inflammatory response initiating the production of anti-inflammatory cytokines, like IL-10 [2,14]. In the current study, a time-by-condition interaction was observed for IL-6 and IL-10. Both IL-6 and IL-10 concentrations were greater with UPF supplementation than PL at 30 min post exercise. Given the role of IL-6 in modulating carbohydrate metabolism, the higher concentrations 30 min post exercise in the UPF condition may suggest a heightened recovery response in an effort to counteract the metabolic demands of HIE. Increased IL-6 coupled with higher concentrations of IL-10 with UPF supplementation, point to a rapid promotion of an anti-inflammatory environment following exercise. While it is noted that large variations in cytokines were observed, the data suggest UPF may play a role in mediating a faster return to homeostasis following intense exercise. Additionally, while there was a lack of significant difference between groups for IL-1β, the overall pattern and magnitude of response following HIE was interesting. Specifically, over the 1-h post-exercise period, a decrease in this pro-inflammatory cytokine was seen with UPF (*d* = -0.16) while a moderate increase was observed for PL (*d* = 0.34). Further consideration may be warranted regarding the impact of sex on this response as males appeared to drive the lowered IL-1β concentrations following supplementation. Given the differences seen in IL-6 and IL-10, and the pattern of IL-1β response during recovery, a longer measurement period may be required to adequately assess these changes.

The inflammatory cytokine response immediately following HIE could be related to the significant changes in WBC, specifically CD4 and CD8 cells, observed with both conditions. Since CD4 cells are cytokine-signaling T-cells [7], a lowered concentration of these WBC acts to help attenuate or mitigate the immediate cytokine response. Induced changes in CD8 cell concentration due to exercise have been observed in studies of greater exercise intensity with longer periods of training in healthy adults [7]. One clinical trial examined changes in T-cell subsets following a WAnT cycling challenge and demonstrated a significant increase in CD8 cell concentration immediately post exercise compared to pre exercise [32]. In the present study, both CD4 and CD8 increased immediately post exercise for both conditions, while CD4 cell concentrations were reduced below baseline levels 60 min post exercise. Inferences regarding inflammation are derived from the measured cytokine and immune cell response following HIE given the study design and measured markers. The addition of macrophages to the blood biomarker profile may have strengthened the assessment of the inflammatory response. However, as macrophages typically do not circulate in the blood and instead reside locally in tissues to facilitate debris clearance following damage [2,33], the need for muscle biopsies limits the feasibility of using this as a marker of inflammation and muscle damage. Further, given the magnitude of biomarker changes in response to the HIE observed in the current study, it may be difficult to elucidate differences with supplementation during the 1-h post HIE recovery period. The nature of the HIE and the profound immune response may have masked potential changes occurring with supplementation. Future studies may involve a longer observation period beyond 1 h.

The secondary hypothesis of improved exercise performance was not supported as no significant PP or MP differences were observed between groups. These results indicate that fucoidans may not act as a direct ergogenic aid to improve performance during acute bouts of HIE. However, unlike the current study, consistently lowered chronic inflammatory markers and improved immune health have been demonstrated in previous research in healthy [19,21], clinical [20], and athletic [27] populations. More research is warranted to determine the chronic effects of fucoidans on facilitating a faster return to play following repeated bouts of intense exercise.

To the authors’ knowledge, this is the first study using fucoidan supplementation as a strategy to impact cytokine and immune cell response following an acute bout of HIE in a human model using a trained population. Importantly, the responses of both males and females were included as a part of this evaluation. As noted, males and females responded similarly to supplementations. However, IL-1β showed a significant sex-by-condition interaction, with no differences seen as a function of UPF supplementation in females. Males experienced higher IL-1β concentrations in the PL condition compared to UPF. Interestingly, there was a sex-by-time interaction for neutrophils by which females experienced greater neutrophil concentrations at timepoint B and D compared to A, whereas males only experienced greater concentrations at timepoint B. The mechanisms behind this interaction may require further investigation, but these findings highlight the importance of considering sex effects in the area of performance nutrition and physiology.

A major strength of the present research is the double-blind, placebo-controlled, counterbalanced, crossover design. All participants partook in both arms of the study with identical procedures for S1/S2 and T1/T2. Additionally, T1/T2 controlled for time of day to account for diurnal rhythms, required no MIE or HIE within 24 h of the testing sessions to avoid superficial inflations of biomarkers, and diet was matched for each participant in the 24 h leading into the testing sessions. Supplementation order was used as a covariate in order to mitigate the effects of familiarization with the HIE protocol between T1 and T2.

However, there are some limitations. Firstly, this research investigated a single acute bout of HIE after 2 weeks of supplementation. Therefore, chronic adaptations and performance changes with supplementation cannot be determined. Future research with longer supplementation periods is warranted to determine the effects on HIE performance and adaptation. Additionally, since the current research investigated the recovery period during a single HIE bout, future research should investigate if fucoidan supplementation could improve recovery status over multiple HIE bouts when rest periods are limited. Importantly, the degree of muscle damage following the HIE bout could not be ascertained by the peripheral inflammatory and immune blood biomarkers assessed in this study. Although this HIE protocol was used previously to induce DOMS and oxidative stress after exercise [29], markers of muscle damage such as creatine kinase or subjective ratings of DOMS were not measured in the current study and may have helped assess how fucoidan may influence EIMD and recovery status. Future research should investigate longer blood collection protocols and local tissue responses following HIE to observe the full range of biomarker changes and better understand how HIE influenced EIMD.

In this study, HIE induced a profound inflammatory and immune system response that persisted throughout the 1-h post-exercise evaluation period. Supplementation with UPF resulted in limited differences in biomarker response with the exception of cytokines. Both IL-6 and IL-10 increased compared to placebo during the 30-min recovery period. Though not statistically significant, the pattern of decreased IL-1β concentrations by 1-h post exercise with supplementation compared with increases seen with PL may indicate a greater anti-inflammatory environment occurring with UPF. Given the differential changes in cytokines occurring during the recovery period, longer supplementation periods (>2 weeks) along with a longer duration of measurement following HIE may be warranted to see additional effects.
